# The Sesquiterpene Lactone-Rich Fraction of *Inula helenium* L. Enhances the Antitumor Effect of Anti-PD-1 Antibody in Colorectal Cancer: Integrative Phytochemical, Transcriptomic, and Experimental Analyses

**DOI:** 10.3390/cancers15030653

**Published:** 2023-01-20

**Authors:** Jaemoo Chun, Sang-Min Park, Minsung Lee, In Jin Ha, Mi-Kyung Jeong

**Affiliations:** 1 KM Convergence Research Division, Korea Institute of Oriental Medicine, Daejeon 34054, Republic of Korea; 2 College of Pharmacy, Chungnam National University, Daejeon 34134, Republic of Korea; 3 Korean Medicine Clinical Trial Center (K-CTC), Kyung Hee University Korean Medicine Hospital, Seoul 02454, Republic of Korea

**Keywords:** *Inula helenium* L., sesquiterpene lactone, colorectal cancer, transcriptome, immunotherapy, tumor microenvironment

## Abstract

**Simple Summary:**

Alantolactone and isoalantolactone—active sesquiterpene lactones of *Inula helenium* L.—have been reported to suppress tumor growth and modulate immune function; however, the potential for these compounds to regulate cancer immunity is unknown. In this study, a combination of phytochemical, transcriptomic, and experimental analyses was used to identify the potential target of active *I. helenium* compounds in colorectal cancer. Our integrative analysis demonstrated that the sesquiterpene lactone-rich fraction of *I. helenium* (SFIH) significantly enhanced the antitumor effect of anti-PD-1 antibody by reducing tumor growth and increasing the survival time of mice. Specifically, SFIH combined with anti-PD-1 antibody significantly increased the proportion of cytotoxic T lymphocytes and M1-like macrophages. Pathway enrichment analysis revealed that combination therapy activated immune-related pathways to a greater extent than monotherapy. Our results provide a paradigm to identify the SFIH therapy in combination with immune checkpoint inhibitors as an integrative perspective of drugs, targets, and pathways.

**Abstract:**

Treatment strategies combining immune checkpoint inhibitors with sesquiterpene lactones have attracted much attention as a promising approach for cancer treatment. We systemically analyzed gene expression profiles of cells in response to two major sesquiterpene lactones, alantolactone and isoalantolactone, and determined whether the sesquiterpene lactone-rich fraction of *Inula helenium* L. (SFIH) enhances the antitumor effect of anti-PD-1 antibody in MC38 colorectal cancer-bearing mice. Gene expression and pathway analysis using RNA sequencing data were used to identify the SFIH-driven combined activity with anti-PD-1 antibody. The results showed that SFIH significantly enhanced the antitumor effect of anti-PD-1 antibody by reducing tumor growth and increasing the survival time of mice. Specifically, SFIH exhibited antitumor activity when combined with anti-PD-1 antibody, and the effects were further enhanced compared with monotherapy. An analysis of immune cells indicated that combination treatment with SFIH and anti-PD-1 antibody significantly increased the proportion of CD8^+^ T cells. Moreover, combination treatment enhanced antitumor immunity by decreasing the population of myeloid-derived suppressor cells and increasing the number of M1-like macrophages. Pathway enrichment analysis revealed that combination therapy activated immune-related pathways to a greater extent than monotherapy. In conclusion, our integrative analysis demonstrates that SFIH enhances the response of murine tumors to anti-PD-1 antibody. These findings provide insight into developing integrative therapeutics and molecular data for the use of natural products as an adjunct treatment for colorectal cancer.

## 1. Introduction

Colorectal cancer (CRC) is the third leading cause of mortality worldwide and is a major public health problem. There were 1.93 million newly diagnosed CRC cases and 0.94 million CRC-associated deaths in 2020, which accounts for 10% and 9.4% of cancer incidence and deaths, respectively [1]. Standard treatment for CRC includes surgery, radiotherapy, chemotherapy, and targeted therapy [2]. However, despite improvements in CRC treatment and the favorable responses occurring in early-stage patients, these treatments are not very effective in advanced-stage patients, who experience metastasis and recurrence and have a poor 5-year survival rate [3]. Therefore, the development of novel treatments is needed to improve the outcome of advanced-stage CRC patients. Recently, immunotherapy with immune checkpoint inhibitors (ICIs), represented by anti-PD-L1 and anti-PD-1, have been successfully used for the treatment of various metastatic cancers including melanoma, lung, head and neck, bladder, and CRC with high microsatellite instability (MSI) [4]. However, CRC patients with microsatellite stability (MSS), which represents the majority of CRC cases, do not respond to immunotherapy [5]. Recently, an immunosuppressive microenvironment was considered to play a role in the response of CRC with MSS to immunotherapy [6]. The absence of immune-activating cells, an increase of immunosuppressive cells, and an abnormal tumor extracellular matrix may create an immunosuppressive microenvironment that eventually results in the failure of ICI therapy [7]. Therefore, it is necessary to develop effective therapeutic strategies to circumvent this limitation and enhance the efficacy of ICI treatment.

Sesquiterpene lactones are naturally occurring secondary metabolites. They are abundant in plant-derived natural products and are primarily found in the Asteraceae family [8]. They are characterized by the presence of an α-methylene-γ-lactone functional group, which readily reacts with nucleophiles within cells [9]. Because of their structural diversity, sesquiterpene lactones exhibit a wide range of biological activities including anti-inflammatory, anticancer, and immunomodulatory effects [10]. Recently, they have gained interest in the field of cancer immunity. Several studies demonstrated that sesquiterpene lactones, such as atractylenolide I [11], artemisinin [12], and mecheliolide [13], regulate cancer immunity and enhance the response to ICI therapy, suggesting that they may be useful immunotherapeutic agents. As sesquiterpene lactones are the source for the development of anticancer drugs, the beneficial roles of plant-derived natural products containing sesquiterpene lactones have become evident in cancer-related diseases. However, there have been few studies related to cancer immunity. In addition, the observed therapeutic effects may occur from components other than the sesquiterpene lactones, making it difficult to clarify the pharmacological effects of sesquiterpene lactones in an unpurified form.

*Inula helenium* L. (Elecampane), a member of the Asteraceae family, is native to Southern and Eastern Europe with widespread distribution in North America and Asia [14]. This plant is rich in sesquiterpene lactones, primarily alantolactone and isoalantolactone [15], which exhibit anticancer activity, including inhibition of drug resistance [16] and metastasis [17] as well as the induction of apoptosis [18]. However, studies examining the immune regulatory effects of *I. helenium* in cancer are limited. Therefore, in the present study, we determined the effect of *I. helenium* on enhancing the efficacy of immunotherapy and its underlying mechanism.

## 2. Materials and Methods

### 2.1. Chemicals and Reagents

Dulbecco’s modified Eagle’s medium (DMEM) and fetal bovine serum (FBS) were obtained from Gibco (Grand Island, NY, USA). Matrigel was purchased from Corning, Inc. (Corning, NY, USA). Anti-mouse PD-1 antibody (clone RMP1-14) and isotype control IgG2a antibody were obtained from BioXCell (West Lebanon, NH, USA). Anti-CD3 (#ab16669) and anti-TGF-β1 (#ab215715) antibodies were purchased from Abcam (Cambridge, MA, USA). Anti-CD8 (#98941) and anti-F4/80 (#70076) antibodies were purchased from Cell Signaling (Beverly, MA, USA). Anti-granzyme B antibody (#14-8822-82) was purchased from Invitrogen (Carlsbad, CA, USA). PE/Cy7 anti-CD3 (#552774), BV605 anti-CD8 (#563152), BV711 anti-CD4 (#563050), APC anti-CD335 (NKp46) (#137608), APC/Cy7 anti-CD45 (#103116), BV421 anti-CD11b (#562605), APC anti-GR-1 (#108412), PE/Cy7 anti-F4/80 (#123114), Alexa488 anti-MHC Class II (#107616), PE anti-CD206 (#141706), and TruStain FcX™ PLUS (#156604) antibodies were purchased from Biolegend (San Diego, CA, USA).

### 2.2. Preparation of the Sesquiterpene Lactone-Rich Fraction of I. helenium (SFIH)

The root of *I. helenium* was purchased from Naemome Dah (Ulsan, Korea), extracted with methanol for 12 h, and sonicated for 2 h at room temperature. The methanol extract was filtered, evaporated under reduced pressure, suspended in distilled water, and partitioned with *n*-hexane to obtain a hexane fraction. The hexane fraction was concentrated at room temperature using a rotary evaporator under vacuum for the efficient and gentle removal of solvent from the fraction. We designated this product SFIH. SFIH was dissolved in 1% DMSO + 1% Tween 80 in saline for in vivo studies.

### 2.3. Qualitative and Quantitative Analysis of Major Sesquiterpene Lactones in SFIH

**Figure 1:**
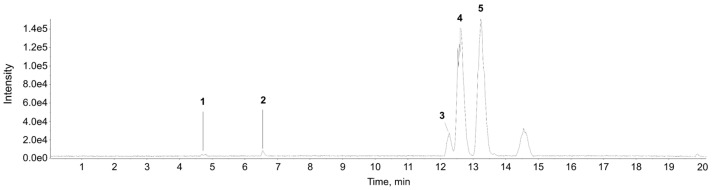
The base peak chromatogram of the sesquiterpene lactones-rich fraction of *Inula helenium* L. (SFIH). The representative base peak chromatogram of SFIH was obtained using LC-ESI-QTOF MS/MS analysis in negative ion mode. 1: Saussureamine A, 2: Parthenolide, 3: Igalan, 4: Isoalantolactone, 5: Alantolactone.

Chromatographic analysis of SFIH was done to identify specific sesquiterpene lactones, including the quantitation of alantolactone and isoalantolactone. SFIH was shaken with ethanol at 20 mg/mL using a vortex mixer for 30 s and sonicated for 10 min. The supernatants were filtered through a 0.2 μm hydrophilic polytetrafluoroethylene syringe filter (Thermo Fisher Scientific, Sunnyvale, CA, USA). Finally, the filtrate was diluted to 0.5 mg/mL and transferred to an LC sample vial for analysis. Chromatographic separation followed by diode array detector and mass spectrometry analysis was done using a Thermo Scientific Vanquish UHPLC system (Thermo Fisher Scientific) with an Acquity UPLC HSS T3 column (2.1 mm × 100 mm, 2.7 μm; Waters, Milford, MA, USA) coupled to a TripleTOF 5600^+^ mass spectrometer (QTOF MS/MS, SCIEX, Foster City, CA, USA). The QTOF MS was equipped with an electrospray ionization (ESI) source in positive ion mode to complete the high-resolution experiment. The elution program for UHPLC separation included 0.1% formic acid in water as eluent A and methanol as eluent B as follows: 0–7 min; 20–50% B, 7–15 min; 50% B, 15–20 min; 50–100% B, 20–23.5 min; 100% B, and equilibration with 20% B for 4 min at a flow rate of 0.4 mL/min. The column temperature was at 25 °C and the auto-sampler was maintained at 4 °C. The injection volume of each sample solution was 2 μL. Data acquisition and processing for qualitative analysis were carried out using Analyst TF 1.7, PeakVeiw 2.2, and MasterView (SCIEX, Foster City, CA, USA). The MS/MS data for qualitative analysis were processed using PeakView 2.2 and MasterView 1.1 to identify putative metabolites based on accurate mass and isotope distribution. The identification of sesquiterpene lactones in SFIH was done using UPLC-ESI-QTOF MS/MS in positive ion mode and concurrent quantitative determination of alantolactone and isoalantolactone in SFIH was done using UPLC-UV at 210 nm. The representative base peak chromatogram of SFIH is shown in Figure 1. Six sesquiterpene lactones were identified in SFIH. Isoalantolactone (4) and alantolactone (5) were identified by comparison with the retention time and fragmentation patterns of reference standards. Saussureamine A (1), parthenolide (2), and igalan (3) were detected and putatively identified by matching the MS/MS fragment ions with our in-house MS/MS library, the GNPS online database, and previous reports [19,20] (Table 1). Among the sesquiterpene lactones, the two major components, isoalantolactone and alantolactone, were measured using regression equations of the calibration curve with a correlation coefficient (*R*^2^) >0.999 (Table 2 and Figure S1). The calibration curves plotted for the concentration range of 5–500 μg/mL for isoalantolactone and 1–500 μg/mL for alantolactone in SFIH are also presented. Finally, isoalantolactone and alantolactone content were determined as 322.62 ± 0.64 and 256.71 ± 0.44 mg/g, respectively.

### 2.4. Microarray Data Analysis

The gene expression profiles induced by alantolactone and isoalantolactone treatment were obtained from the microarray data generated from the MCF-7 cell line [21]. The gene expression profiles were analyzed using the *limma* package (v.3.52.2) in R (v.4.2.1) after normalization by multi-array average. For each phytochemical, differentially expressed genes were selected by thresholds of *p*-values < 0.05 and a fold-change > 1.5. Pathway enrichment analysis was performed using the *enrichR* package (v.3.1). This method identifies biological pathways that are statistically overrepresented more than would be expected by chance in a gene list. The gene set of “BioPlanet_2019”, which is available from the National Center for Advancing Translational Sciences (NCATS) of the National Institutes of Health and consists of 1510 distinct pathways encompassing 9813 genes [22], and the gene set of “KEGG_2021_Human”, which is available from the Kyoto Encyclopedia of Genes and Genomes (KEGG) and consists of 320 distinct pathways encompassing 8078 genes, were used for pathway enrichment analysis.

### 2.5. Animals

Specific pathogen-free 6-week-old female C57BL/6 mice were purchased from Saeron Bio, Inc. (Uiwang, Republic of Korea) and acclimated for 1 week. The mice were housed under controlled environmental conditions of temperature (23 °C), humidity (50%), and a 12-h light/dark cycle. The animals were fed a standard chow diet and provided free access to drinking water. All experimental procedures were approved by the Animal Care and Use Committee of the Korea Institute of Oriental Medicine (Approval number: 21-035).

### 2.6. Cell Culture

MC38 cells were purchased from ABM (Richmond, BC, Canada). MC38 cells were grown in DMEM supplemented with 10% FBS, 100 U/mL penicillin, and 100 μg/mL streptomycin at 37 °C in a humidified atmosphere containing 5% CO_2_.

### 2.7. Tumor-Bearing Mice and Treatment

Tumor-bearing mice were established by injecting 1 × 10^5^ MC38 cells into the right flank subcutaneously of C57BL/6 mice. When the tumor volume reached >50 mm^3^, the mice were divided into four groups (*n* = 7/group) and intraperitoneally injected with 50 mg/kg SFIH daily and/or 200 μg PD-1 antibody two times a week. Control mice were injected with the same volume of saline containing 1% DMSO and 1% Tween 80 and IgG2a antibody. Tumor volume was measured 2 to 3 times a week using calipers and calculated by the following formula: length × width^2^ × 1/2. The body weight and survival of mice were monitored during the experiments. Tumors were dissected, weighed, and dissociated for subsequent analysis.

### 2.8. Immunohistochemistry (IHC)

Tumor tissues were fixed in 10% neutral-buffered formalin (Sigma-Aldrich) for 2 days and processed into paraffin blocks. Paraffin-embedded sections (4-μm thick) were boiled in Tris–EDTA antigen retrieval buffer and incubated in 3% H_2_O_2_ for 10 min. Nonspecific antibody binding was blocked using Super Block (AAA125, ScyTek, UT, USA). The slides were then incubated overnight at 4 °C with primary antibody, including CD3 (1:500), CD8 (1:800), granzyme B (1:300), F4/80 (1:600), TGF-β1 (1:500), and Ki67 (1:600). The following steps were performed using the Polink-2 HRP Plus Rabbit DAB Detection System (GBI Labs, Bothell, WA, USA). The stained slides were cover-slipped using Leica MM24 mounting medium (Leica Biosystems, Richmond, IL, USA) and scanned using Pannoramic DESK (3DHistech, Budapest, Hungary).

### 2.9. Flow Cytometry Analysis

Immune population analysis was done by flow cytometry. Tumor tissues were dissociated using a mouse tumor dissociation kit and a gentle MACS Dissociator (Miltenyi Biotec, Bergisch Gladbach, Germany). Spleens were dissociated by gentle grinding with a syringe plunger. The cells were treated with RBC lysis buffer (Biolegend), suspended in a cell staining buffer (Biolegend), and blocked by nonspecific staining with TruStain FcX™ PLUS. The cells were then incubated for 30 min (4 °C in the dark) with fluorescence-conjugated antibodies (1:100) specific for the lymphocyte markers anti-CD45, anti-CD3, anti-CD8, anti-CD4, and anti-NKp46, and the monocyte markers anti-CD45, anti-CD11b, anti-GR-1, anti-F4/80, anti-MHC Class II, and anti-CD206. Finally, the cells were analyzed using a BD LSRFortessa™ X-20 flow cytometer (BD Biosciences, San Jose, CA, USA) with FlowJo software (BD Biosciences).

### 2.10. RNA Sequencing Data Analysis

Total RNA was isolated from tumor tissues using TRIzol (Invitrogen) following the manufacturer’s protocol. The RNA quality was assessed using a Bioanalyzer 2100 System (Agilent Technologies, Santa Clara, CA, USA). RNA concentration was measured using a NanoDrop ND-2000 (Thermo Fisher Scientific). The cDNA libraries were constructed using the QuantSeq 3′ mRNA-Seq Library Prep Kit (Lexogen, Inc., Vienna, Austria) and sequenced on a NextSeq 500 (Illumina Inc., San Diego, CA, USA) to generate single-end 75 bp reads according to the manufacturer’s instructions. From the raw count, transcripts per million (TPM) were calculated to determine gene expression levels in R (v.4.2.1). Clustering analysis with t-distributed stochastic neighbor embedding (tSNE) was performed with the *Rtsne* package (v.0.16). Principal component analysis (PCA) was performed with the *DESeq2* package (v.1.36). Gene set enrichment analysis (GSEA) was done using the *fgsea* package and the hallmark gene set and Gene Ontology (GO) terms from the Molecular Signature Database (MSigDB). GSEA results for the GO terms were summarized and visualized in a simplified network by the EnrichmentMap application (v.3.3.4) in Cytoscape (v.3.9.1).

### 2.11. Statistical Analysis

Statistical analysis was performed using Graphpad Prism 8 software. Data with error bars represent the mean ± standard deviation (SD), except for tumor volume and weight, which represents the mean ± standard error of the mean (SEM). Statistical analysis of significance was based on a two-tailed Student’s *t* test. Animal survival was determined using Kaplan–Meier survival curves and analyzed by the Gehan–Breslow–Wilcoxon test. A value of *p* < 0.05 was considered statistically significant. Statistical analysis of transcriptome data of microarray and RNA sequencing was performed using R packages *limma*, *enrichr*, *DESeq2*, and *fgsea* for differentially expressed genes (DEGs), pathways enrichment analysis, and GSEA. Adjusted *p*-values (*padj*) were obtained from *p*-values by multiple testing corrections via false discovery rate (FDR) estimation.

## 3. Results

### 3.1. Identification of SFIH Target Genes and Pathway Enrichment Analysis

**Figure 2:**
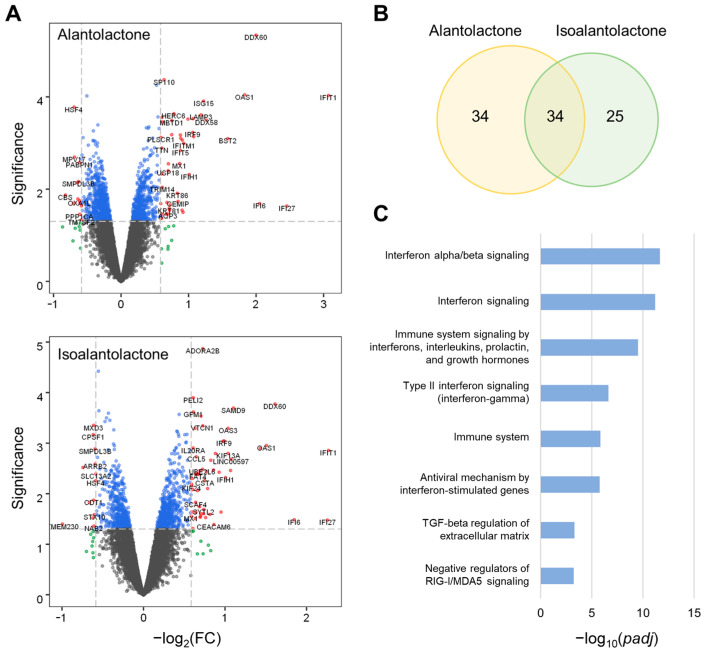
Predicted target pathways of alantolactone and isoalantolactone. (**A**) Volcano plots for the gene expression profiles in response to treatment with alantolactone or isoalantolactone in MCF-7 cells. The significance was calculated by −log_10_(*p*-value). DEGs were selected with threshold *p* < 0.05 and |FC| > 1.5. (**B**) Venn diagram for the number of differentially expressed genes (DEGs) from alantolactone and isoalantolactone treatment. (**C**) Significantly enriched pathways (*padj* < 0.01) associated with the DEGs of the two phytochemicals by *enrichR* using BioPlanet_2019. FC, fold change; *padj*, adjusted *p*-value.

Transcriptomics provides powerful data for analyzing the biological effects of small molecules [23]. To identify target pathways regulated by SFIH, we first examined the key ingredients of SFIH, which consist predominantly of alantolactone and isoalantolactone. Because studies of known targets of alantolactone and isoalantolactone are limited, we analyzed the genome-wide effects of these two molecules using the drug-induced transcriptome dataset generated by treatment of MCF-7 cells with phytochemicals obtained from herbal medicines [21]. From the data involving the response to alantolactone and isoalantolactone treatment, we identified a set of DEGs (Figure 2A). The DEGs for the two molecules overlapped one another (Figure 2B). Pathway enrichment analysis for the combined DEGs using the BioPlanet gene set indicated that several immune-related pathways, including interferon signaling, were significantly associated with alantolactone and isoalantolactone treatment (Figure 2C). The activation of interferon signaling has a crucial role in regulating the tumor microenvironment to provide favorable conditions for PD-1 blockade [24,25]. Pathway enrichment analysis using the KEGG gene set for the combined DEGs focused on immune-related diseases (Figure S2). In addition, the DEGs of the two molecules were also associated with the transforming growth factor (TGF)-β signaling pathway, which impairs antitumor immune response such as antigen presentation and T cell infiltration in the tumor microenvironment, causing a limited response to ICIs [26,27]. These results indicate that SFIH may contribute to a better response to ICI treatment by modulating immune-activating or immunosuppressive conditions in the tumor microenvironment.

### 3.2. SFIH Combined with Anti-PD-1 Antibody Suppresses Tumor Growth and Results in Improved Survival of MC38 Tumor-Bearing Mice

**Figure 3:**
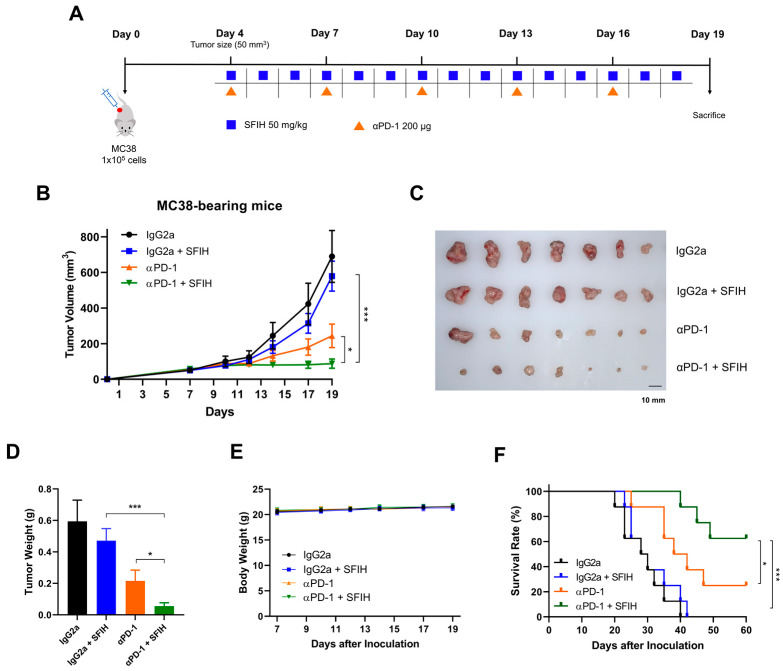
SFIH enhances the antitumor effect of anti-PD-1 antibody in MC38 colorectal tumor. (**A**) Schematic diagram of SFIH and anti-PD-1 antibody treatment. A total of 1 × 10^5^ MC38 cells were subcutaneously injected into C57BL/6 mice. When the tumor volume reached >50 mm^3^, the mice were divided into four groups (*n* = 7/group) as follows: IgG2a, IgG2a with SFIH, αPD-1, and αPD-1 with SFIH. SFIH (50 mg/kg) was intraperitoneally administered daily. Anti-PD-1 antibody (200 μg) was intraperitoneally administered every 3 days. (**B**) Tumor volume changes in MC38-bearing mice. Tumor growth was monitored every 2 or 3 days by measuring tumor volume. Error bars represent SEM. (**C**) MC38 tumors were separated and photographed. (**D**) Tumor weights are shown. Error bars represent SEM. (**E**) Body weight changes of MC38-bearing mice. Error bars represent SD. (**F**) Kaplan–Meier survival distribution in the model is displayed for the mice following treatment (*n* = 8/group). *p*-values were determined using the two-tailed Student’s *t* test for tumor volume and tumor weight and the Gehan–Breslow–Wilcoxon test for the survival curve (* *p* < 0.05 and *** *p* < 0.001, compared to the combination group).

The above findings prompted an in vivo study to evaluate the immunomodulatory effects of SFIH combined with PD-1 blockade in the tumor microenvironment. To determine whether SFIH exerts a synergistic effect on the antitumor immune response of an anti-PD-1 antibody, C57BL/6 mice were subcutaneously implanted with MC38 cells. SFIH and/or anti-PD-1 antibody were then administered daily and twice a week, respectively (Figure 3A). Tumor growth was not significantly affected by SFIH monotherapy, indicating that SFIH alone is ineffective; however, treatment with anti-PD-1 antibody resulted in a significant reduction of tumor volume, whereas a marked reduction was observed with SFIH plus anti-PD-1 (Figure 3B,C), suggesting that SFIH confers immune remodeling to inhibit tumor growth. MC38 tumor weight was also further decreased in the combination group (Figure 3D). No significant decrease was observed in body weight in either group (Figure 3E). Next, we determined the effect of combined treatment on overall survival. The control group survived for a median of 29 days, whereas the SFIH group did not survive any longer. However, the combination treatment group survived significantly longer compared with the anti-PD-1 (median 40 days, *p* < 0.05) and SFIH (median 29 days, *p* < 0.001) groups, suggesting that it is significantly more effective than either treatment as monotherapy (Figure 3F). Taken together, these results indicate that SFIH enhances the response of anti-PD-1 antibody and may contribute to antitumor immunity in MC38-bearing mice.

### 3.3. SFIH Combined with Anti-PD-1 Antibody Promotes the Infiltration of Immune Cells

**Figure 4:**
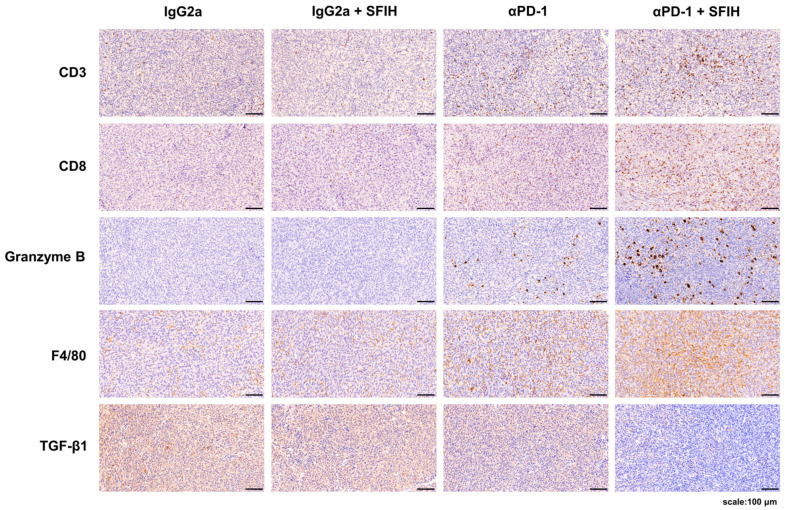
SFIH combined with an anti-PD-1 antibody alters the immune landscape in the tumor microenvironment. Immunohistochemistry (IHC) staining of MC38 tumors for infiltration of immune cells. CD3, CD8, granzyme B, F4/80, and TGF-β1 levels were assessed by microscopic examination of IHC-stained sections. Representative IHC-stained images are shown (scale bars = 100 µm).

To identify immune population differences following combined treatment with SFIH and anti-PD-1 antibody, IHC was performed. As shown in Figure 4, SFIH monotherapy did not affect CD3^+^ and CD8^+^ T cell infiltration and granzyme B levels, although they were increased slightly by anti-PD-1 antibody monotherapy. However, combination treatment resulted in a marked infiltration of CD3^+^ and CD8^+^ T cells and increased the release of granzyme B compared with monotherapy. Furthermore, IHC staining confirmed that combination treatment increased the proportion of macrophages compared with monotherapy. Combination treatment decreased the expression of TGF-β1, which is associated with an immunosuppressive environment, leading to ICI resistance [28]. The correlation of IHC staining intensity and tumor inhibition percentage was performed by Pearson’s correlation coefficient method. The results showed that tumor inhibition positively correlated with CD8, granzyme B, and F4/80, but was inversely linked with TGF-β1 in tumor tissues (Figure S3). Taken together, these results suggest that SFIH combined with anti-PD-1 antibody may promote the infiltration and activity of CD8^+^ T cells and regulate macrophage differentiation in tumor tissues, thereby enhancing tumor response to anti-PD-1 antibody.

### 3.4. SFIH Combined with Anti-PD-1 Antibody Enhances the Antitumor Immune Response by Cytotoxic T Lymphocytes and M1-Like Macrophages

**Figure 5:**
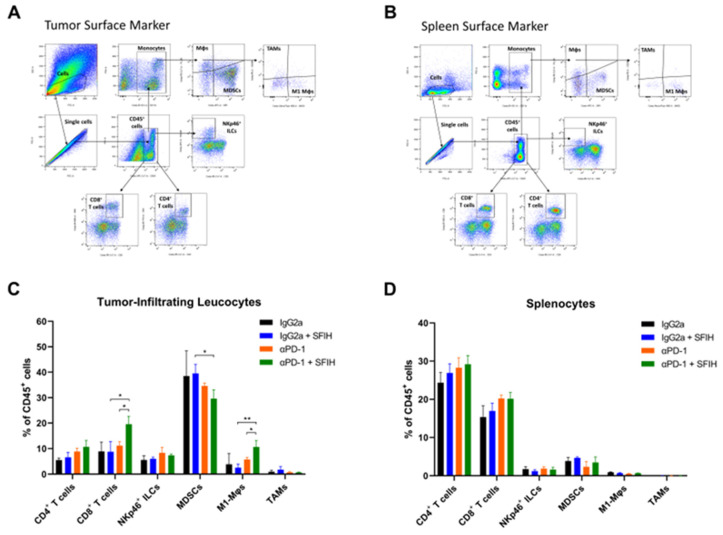
SFIH combined with an anti-PD-1 promoted antitumor immune response of cytotoxic T lymphocytes and M1-like macrophages in MC38 tumor-bearing mice. (**A**,**B**) Gating strategy for flow cytometry analysis of tumor-infiltrating CD45^+^ leucocytes in tumor tissues (**A**) and spleens (**B**). (**C**,**D**) The subset of tumor-infiltrating lymphocytes, including T cells and NKp46^+^ ILCs, and tumor-infiltrating monocytes, including MDSCs, M1-like macrophages, and TAMs in tumor tissues (**C**) and spleens (**D**). Error bars represent SD. *p*-values were determined using a two-tailed Student’s *t* test (* *p* < 0.05 and ** *p* < 0.01, compared to the combination group). ILCs, innate lymphoid cells; M1-Mφs, M1-like macrophages; TAMs, tumor-associated macrophages; MDSCs, myeloid-derived suppressor cells.

To further explore the immune landscape in MC38 tumor-bearing mice, we analyzed tumor-infiltrating leucocytes (CD45^+^ cells) using flow cytometry in tumor tissues (Figure 5A) and spleens (Figure 5B). SFIH or anti-PD-1 antibody monotherapy did not affect the population of CD8^+^ T cells; however, combined treatment significantly increased the population of CD8^+^ T cells in tumor tissues, which was consistent with the results of IHC. The population of NKp46^+^ innate lymphoid cells (ILCs) showed no differences between the treatment groups. In addition, combined treatment resulted in a decreased population of immunosuppressive myeloid-derived suppressor cells (MDSCs) (CD11b^+^, GR^+^). We further examined the proportion of M1-like macrophages and tumor-associated macrophages (TAMs) to identify the subtype of the macrophages. Combined treatment resulted in an increased population of M1-like macrophages (CD11b^+^, F4/80^+^, MHC Class II^+^, CD206^−^), but did not change the population of TAMs (CD11b^+^, F4/80^+^, MHC Class II^−^, CD206^+^) in tumors (Figure 5C). We confirmed the effect of combined treatment on the systemic immune responses; however, no significant changes were observed in the lymphocyte and monocyte populations between treatment groups (Figure 5D). Collectively, these results suggest that combination treatment of SFIH and anti-PD-1 antibody increases M1-like macrophages in the tumor microenvironment and causes the infiltration of CD8^+^ T cells, thereby enhancing the antitumor effects.

### 3.5. SFIH Combined with Anti-PD-1 Antibody Further Enhanced Antitumor Immune-Related Pathways

**Figure 6:**
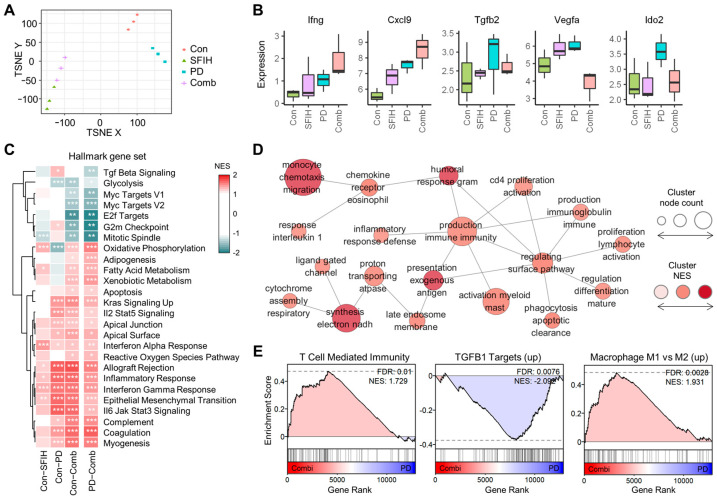
Transcriptomic analysis of combined treatment with SFIH and anti-PD-1. (**A**) Cluster analysis with the gene expression data from the four mouse groups. (**B**) Expression levels of five selected genes in the four mouse groups. The levels were calculated as log_2_ (TPM + 1). (**C**) GSEA results using the hallmark gene set. Normalized enrichment score (NES) values are indicated by colors and adjusted *p*-values are denoted by asterisks as follows: * *padj* < 0.05, ** *padj* < 0.01, and *** *padj* < 0.005. Only results with significant changes in the combined treatment compared with the anti-PD-1 treatment (“PD-Comb”) are shown. (**D**) The largest increased component in the simplified network from the GSEA results of PD-Comb using the GO terms. Each node represents a cluster of integrated similarly enriched terms, named with common keywords for the terms included. (**E**) The GSEA results of PD-Comb for “GOBP_T_CELL_MEDIATED_IMMUNITY” (left), “KARLSSON_TGFB1_TARGETS_UP” (middle), and “COATES_MACROPHAGE_M1_VS_M2_UP” (right). Con, control group; SFIH, SFIH treatment group; PD, anti-PD-1 treatment group; Comb, SFIH + anti-PD-1 antibody treatment group.

To further examine the underlying mechanisms for the enhanced antitumor effect of the combination, an RNA sequencing analysis was done using the MC38 tumor tissues from the four treated groups: control (denoted as “Con”), SFIH monotherapy, anti-PD-1 antibody monotherapy (denoted as “PD”), and combined treatment (denoted as “Comb”). Cluster analysis revealed that each of the four groups had distinct expression profiles (Figure 6A and Figure S4). We found that Ifng and Cxcl9 were increased by the combined treatment, whereas Tgfb2, Vegfa, and Ido2 were decreased (Figure 6B). GSEA was then performed for the three treatment conditions compared with the control, whereas the combined treatment was compared to anti-PD-1 antibody monotherapy using the hallmark gene set. The results indicated that apoptosis, interferon-gamma response, and inflammatory response were significantly increased following combined therapy, whereas TGF beta signaling was significantly decreased (Figure 6C). To highlight differences in pathway activity of the combined treatment compared with the anti-PD-1 antibody monotherapy, we performed GSEA using the GO terms. The hundreds of enriched terms associated with the combined treatment are represented in a simplified network by EnrichmentMap, which integrates similar terms into a single node with common keywords (Figure S5). The largest connected component of increased terms in the simplified network was primarily focused on the immune response, such as lymphocyte proliferation and activation, antigen presentation, immunoglobulin production, interleukin 1 response, and inflammatory response (Figure 6D). Specifically, the combined treatment was associated with an increase in gene sets involved in T cell activation and T cell-mediated immunity (Figure 6E and Figure S6). For the combined treatment, genes upregulated by TGFB1 were decreased, but genes upregulated in M1 compared with M2 were increased (Figure 6E). These results suggest that the combination treatment modulates macrophage differentiation and facilitates T cell-mediated immunity in the tumor microenvironment. 

## 4. Discussion

The advent of ICI therapy in oncology has revolutionized cancer treatment and enlarged the field of cancer immunology [29]. ICIs, such as pembrolizumab, nivolumab, and atezolizumab, have significantly increased the overall survival rate of many solid cancers, including non-small cell lung cancer, melanoma, and CRC; however, only a limited number of patients benefit from ICI therapy [30]. To improve the survival and response rates, combining various therapeutic agents with ICI therapy has been evaluated in preclinical and clinical trials [31]. Understanding the role of the tumor microenvironment with respect to the antitumor immune response of ICIs is important [32]. For example, an acidic and hypoxic environment affects T cell metabolism and nutrient deprivation in the tumor microenvironment and inhibits T cell proliferation. MDSCs in the tumor microenvironment express high levels of arginase-1, which decreases arginine levels and inhibits the T cell response [33]. Furthermore, there are many immunosuppressive and immune-evading mechanisms in the tumor microenvironment [34]. Therefore, combination therapy targeting the tumor microenvironment by immunomodulation and remodeling the tumor microenvironment with nutrients represents a novel strategy for CRC treatment [35].

Natural products are considered a valuable resource for the prevention and treatment of cancer; however, their mode of action has yet to be established, although several natural products exert powerful antitumor effects [36]. One major problem is the complexity of the components. Because numerous compounds influence the overall pharmacological effect, a comprehensive analysis of the components is required [37]. Studies on the pharmacological effect of natural products have focused only on the entire chemical components of natural products and/or extracts. Because the amount of the individual compounds responsible for pharmacological activity may be limited, screening natural products for active components is challenging. Therefore, it is necessary to identify pharmacological marker compounds and develop techniques to enrich or purify these active compounds.

To address this limitation, we prepared and analyzed a hexane fraction from *I. helenium*, which contains sesquiterpene lactones. Sesquiterpene lactones have long been recognized as a source of anticancer drugs [38]. SFIH consists of several sesquiterpene lactones, including saussureamine A, parthenolide, igalan, alantolactone, and isoalantolactone, which regulate the antitumor immune response of T cells, NK cells, DCs, B cells, and macrophages through multiple signaling pathways. However, the pharmacological effects of SFIH are likely based on the activity of alantolactone and its isomer, isoalantolactone, as their content was 322.62 and 256.71 mg/g, respectively. Interestingly, alantolactone combined with other compounds promoted an antitumor response in CRC in combination with MSS [39].

To determine the potential of SFIH against cancer, we used a systematic strategy to clarify the active components and their mode of action. The network pharmacological approach has been widely used for predicting molecular mechanisms of phytochemicals and herbal medicines; however, this approach requires known target gene information [40]. Therefore, it is limited with respect to the amount of prior knowledge available and its application is limited when information is scarce, such as that for alantolactone and isoalantolactone. On the other hand, a transcriptomic approach primarily focused on drug-induced gene expression datasets provides powerful information for identifying DEGs following treatment with each compound and their interactions that predict a molecular mechanism [41]. In addition, a systems pharmacological approach based on the transcriptome induced by phytochemical and herbal medicine treatment can identify molecular mechanisms in a systematic way [42]. We thought to infer the molecular mechanism of *I. helenium* from the database in advance prior to administration experiments in mouse models. According to a recent study of molecular mechanisms underlying cold and hot properties of herbal medicines, the results of transcriptome-based pathway analysis were comparable to those of target-based pathway analysis [43]. Furthermore, transcriptome-based pathway analysis predicted novel molecular mechanisms for Paeoniae Radix and Bupleuri Radix, which were validated in subsequent experiments [44,45]. In the present study, a systems pharmacological approach was used to identify target pathways of the main sesquiterpene lactones, alantolactone and isoalantolactone, using the phytochemical-induced transcriptome dataset generated in a breast cancer cell line MCF-7 [21]. Previous studies have demonstrated that gene signatures obtained in a specific cell line can reveal common molecular mechanisms across tissues. Using the same dataset, Wang et al. identified that phillyrin reduced the expression of genes associated with cardiac fibrosis and validated the effect of phillyrin as an anti-cardiac fibrosis agent in a rat model of myocardial infarction [46]. In addition, Li et al. identified several vasodilators among the ingredients and validated them in the aorta of a rat model [47]. For another example, as a result of analyzing the gene signature of Bupleuri Radix treated on a lung cancer cell line A549, the effect of promoting cell adhesion and migration was revealed, which was experimentally verified not only in lung cancer cells but also in skin cells [44]. Therefore, the pathways of action of SFIH identified in this study may work beyond breast cancer, including colorectal cancer. Our results showed that pathway enrichment analysis revealed that SFIH regulates antitumor immunity by attenuating IFN-γ signaling and the TGF-β pathway, which have important roles in the response to cancer immunotherapy.

In our in vivo model, we demonstrated that SFIH combined with an anti-PD-1 antibody enhanced antitumor activity and increased survival in MC38 colorectal cancer-bearing mice. Although anti-PD-1 monotherapy caused a delay in tumor growth, combination treatment was more pronounced. Mechanistically, SFIH combined with an anti-PD-1 promoted antitumor immune responses by cytotoxic T lymphocytes and M1-like macrophages. Tumor suppression coincided with an increase of CD8^+^ T cells, granzyme B staining, and M1-like macrophages. In addition, a putative mechanism for the effect of the combination on antitumor immunity was confirmed by RNA sequencing data, which provides a large amount of gene expression data to characterize the tumor microenvironment and identify relevant pathways [48]. Gene expression analysis revealed that the combination treatment was associated with an increase in immune-related factors and a decrease in immunosuppressive factors. In particular, the combined treatment was strongly related to the IFN-γ immune response by lymphocytes. We also confirmed that the upregulated genes in M1 compared with M2 were increased by the combination treatment, indicating that the combination is associated with differentiation into M1-like macrophages. From our integrative analysis, SFIH modulates several target pathways of alantolactone and isoalantolactone including IFN-γ signaling and the TGF-β pathway. These target pathways were verified in experiments with SFIH combined with anti-PD-1 antibody. The results demonstrate that drug-induced transcriptomes can reveal molecular mechanisms underlying the effects of phytochemicals and herbal medicines. There are some limitations in our study. We performed RNA sequencing on bulk tissues. The shortcoming of bulk RNA sequencing is that it only provides an average of gene expression values in a mixture of different cells [49]. In addition, the mouse tumor model does not replicate the tumor microenvironment and immune system of humans. Therefore, further studies will be conducted to examine the efficacy of the combination treatment in humanized NSG mice model which is reconstituted by engraftment of human peripheral blood mononuclear cells. Nevertheless, our mouse tumor model using syngeneic cell lines is very useful in understanding basic concepts of natural products and immunotherapies. Although we did not perform the antibody-mediated depletion of CD8^+^ T cells and macrophages, the depletion of CD8^+^ T cells completely abrogated the anti-PD-1-induced tumor inhibition whereas the depletion of CD4^+^ T cells, B cells, and NK cells does not in MC38 tumor-bearing C57BL/6 mice [50]. In addition, intratumoral CD8^+^ T cells and CD11b^+^ myeloid cells play a crucial role in response to anti-PD-1 antibody treatment in the MC38 tumor-bearing mice [51]. In particular, CD11b^+^ myeloid cells in the tumor microenvironment of responders are polarized toward macrophages with an M1-like signature [52]. Although further studies are needed to examine the detailed mechanism, these findings strongly support the potential of combination therapy through an integrative analysis.

## 5. Conclusions

We systematically identified the role of SFIH involved in cancer immunity by evaluating the key ingredients, target genes, and transcriptomic signatures. Our integrative analysis revealed that SFIH enhances the antitumor activity of anti-PD-1 antibody by increasing the proportion of CD8^+^ T cells and M1-like macrophages in the tumor microenvironment. Overall, this strategy provides a paradigm to identify the role of SFIH in combination with ICIs as an integrative perspective on drugs, targets, and predicted pathways.

## Figures and Tables

**Table 1:** Identification and characterization of chemical components in SFIH using LC-ESI-QTOF MS/MS. RT = retention time.

No.	Compound	Formula	Mass (Da)	Expected RT (min)	Adduct	Found at Mass (Da)	Error (ppm)	MS/MS Product Ions
1	Saussureamine A	C_20_H_29_NO_4_	347.2097	4.80	[M + H] ^+^	348.21717	0.7	302.2118, 128.0708, 100.0767, 82.0664
2	Parthenolide	C_15_H_20_O_3_	248.1412	6.56	[M + H] ^+^	249.14839	−0.5	105.0709, 145.1008, 185.1323, 91.0554
3	Igalan	C_15_H_20_O_2_	232.1463	12.26	[M + H] ^+^	233.15375	0.6	187.1485, 145.1015, 105.0709, 131.0860
4	Isoalantolactone	C_15_H_20_O_2_	232.1463	12.65	[M + H] ^+^	233.15383	0.9	105.0710, 91.0558, 131.0858, 145.1012
5	Alantolactone	C_15_H_20_O_2_	232.1463	13.30	[M + H] ^+^	233.15382	0.9	105.0709, 91.0556, 151.0754, 117.0702

**Table 2:** Calibration parameters and alantolactone and isoalantolactone content in SFIH using LC-UV.

Name	Calibration Parameters			Contents (mg/g)
	Equation for Calibration	*R* ^2^	Linear Range (μg/mL)	
Alantolactone	y = 0.2496x − 0.0876	0.9996	1–500	256.71 ± 0.44
Isoalantolactone	y = 0.1788x − 0.0500	0.9995	5–500	322.62 ± 0.64

## Data Availability

The raw sequence and processed data were deposited in the NCBI Gene Expression Omnibus (GEO, https://www.ncbi.nlm.nih.gov/geo/ (accessed on 30 December 2022)) with accession number GSE221784. The data presented in this study are available on request from the corresponding author.

## Supplementary Materials

The following supporting information can be downloaded at: https://www.mdpi.com/article/10.3390/cancers15030653/s1, Figure S1: UPLC-UV chromatograms from a mixture of reference standards and SFIH; Figure S2: Pathway enrichment analysis using the KEGG gene set; Figure S3: The quantitative results for IHC and the correlation analysis; Figure S4: PCA plot for the gene expression data from the mouse groups; Figure S5: A simplified network for GSEA results with GO terms significantly affected by the combination treatment; Figure S6: GSEA plots with the gene sets significantly affected by the combination treatment.
