# Supplementary material for: The Sesquiterpene Lactone-Rich Fraction of Inula helenium L. Enhances the Antitumor Effect of Anti-PD-1 Antibody in Colorectal Cancer: Integrative Phytochemical, Transcriptomic, and Experimental Analyses
Source: Cancers (Basel). 2023 Jan 20;15(3):653. doi: 10.3390/cancers15030653 (PMC9913754; doi:10.3390/cancers15030653)
Supplement: Supplementary file 1 [file cancers-15-00653-s001.zip › cancers-2066421-supplementary.pdf]

Supplementary Materials: The Sesquiterpene Lactone-Rich Fraction of *Inula helenium* L. Enhances the Antitumor Effect of Anti-PD-1 Antibody in Colorectal Cancer: Integrative Phytochemical, Transcriptomic, and Experimental Analyses

Jaemoo Chun, Sang-Min Park, Minsung Lee, In Jin Ha and Mi-Kyung Jeong

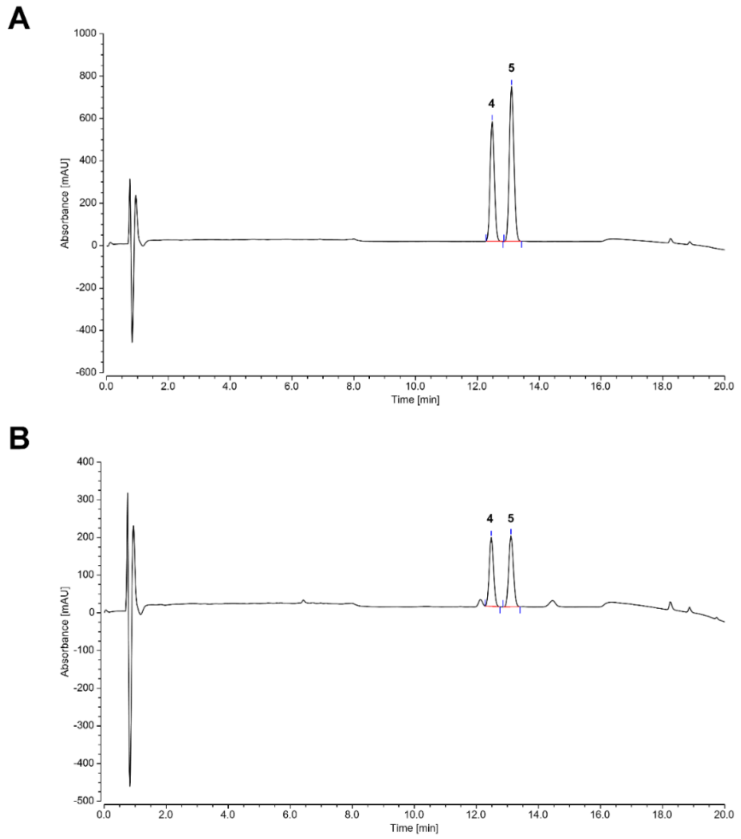

Figure S1. UPLC-UV chromatograms from a mixture of reference standards **(**A**)** and sesquiterpene lactones-rich fraction of *Inula helenium* L. **(**B**)** at 210 nm. 4: Isoalantolactone, 5: Alantolactone.

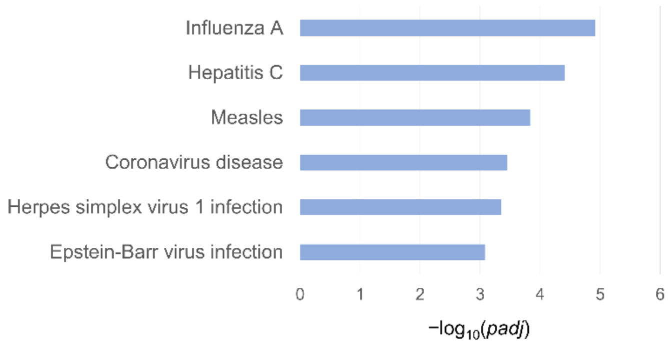

Figure S2. Pathway enrichment analysis using the KEGG gene set for the combined DEGs focused on immune-related diseases.

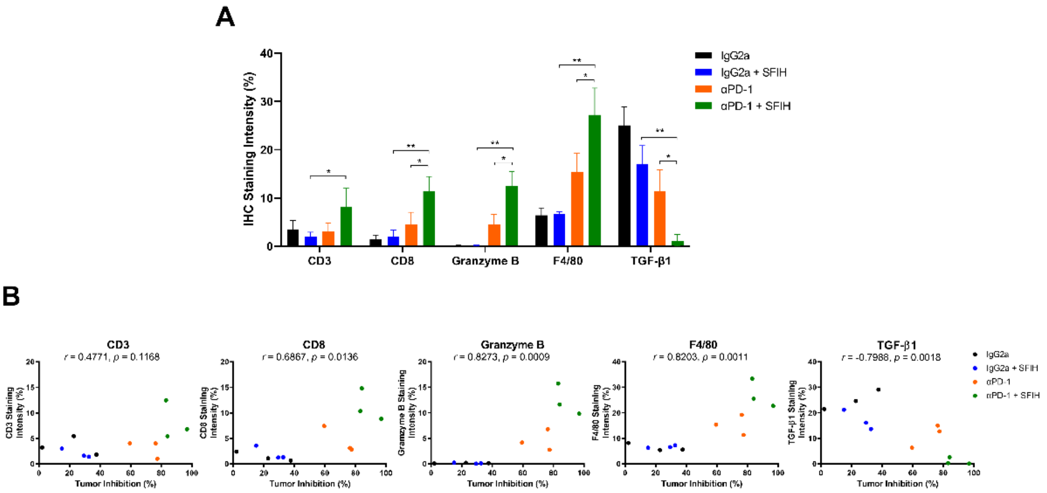

Figure S3. The quantitative results for IHC. **(A)** Quantification analysis of IHC staining was performed using the ImageJ software. Data are represented as the mean ± SD. *p*-values were determined using a two-tailed Student's *t* test (**p* < 0.05 and ***p* < 0.01, compared to the combination group). **(B)** The correlation analysis between IHC staining intensity and tumor inhibition percentage. r represents Pearson's correlation coefficient. Two-tailed *p*-value was determined with Pearson's correlation coefficient.

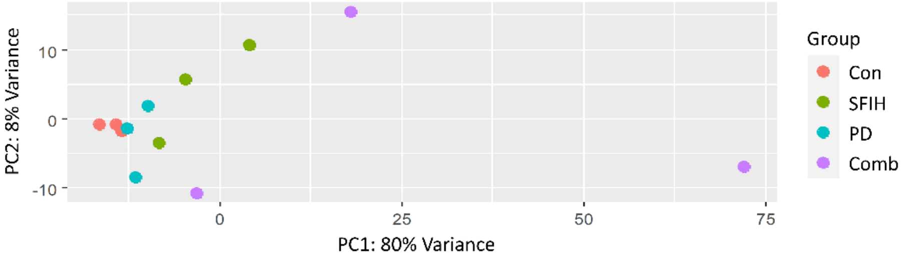

Figure S4. Principal component analysis (PCA) plot for the gene expression data from the mouse groups. PCA was performed with the *DESeq2* package (v.1.36).

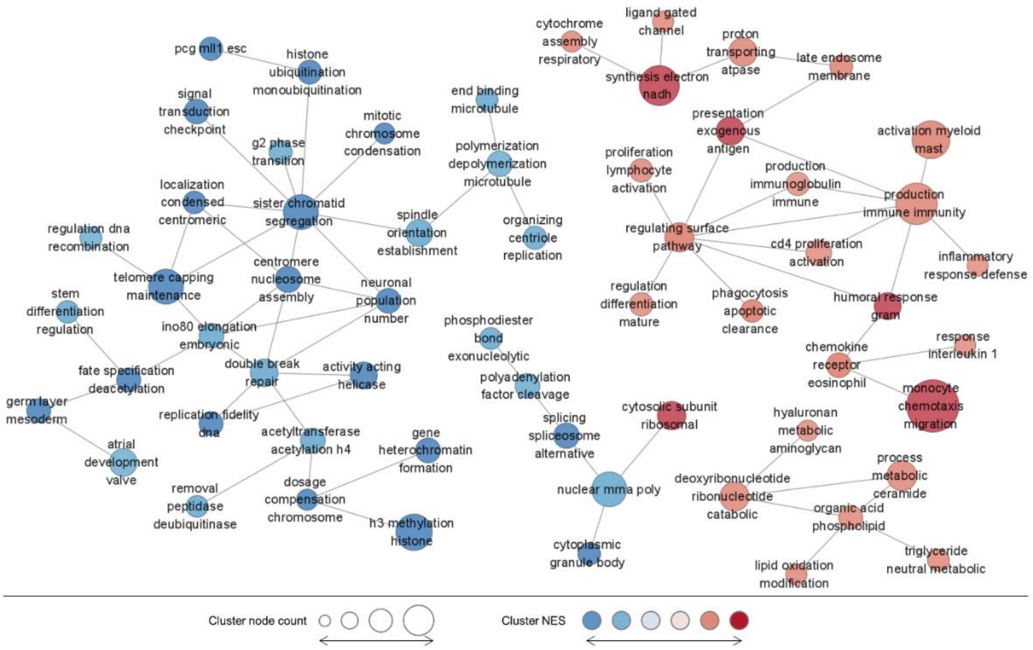

Figure S5. A simplified network for GSEA results with GO terms significantly affected by the combination treatment compared to the anti-PD-1 treatment. The network was reconstructed by EnrichmentMap in Cytoscape.

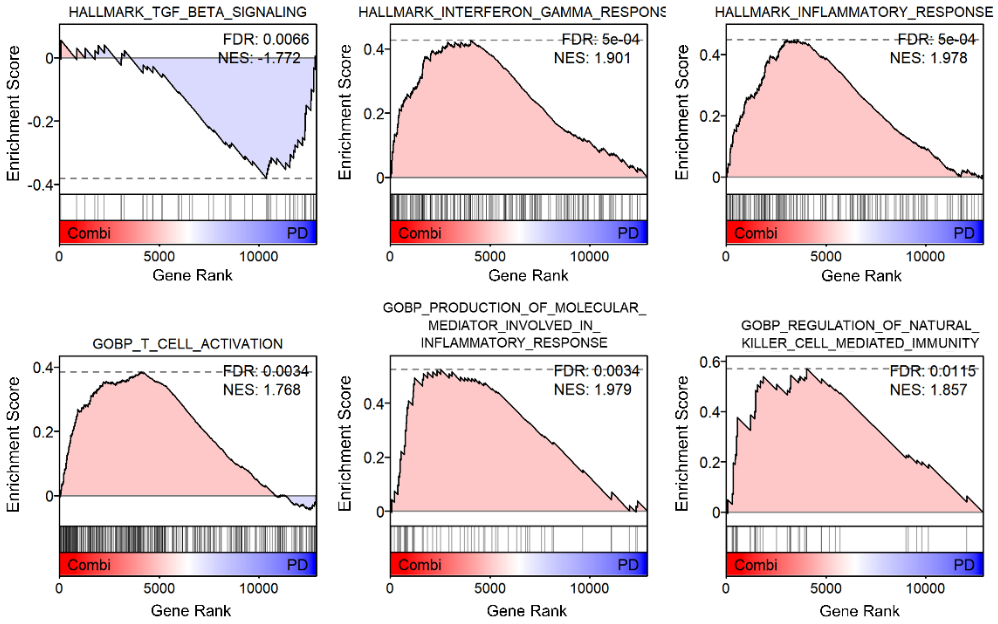

Figure S6. GSEA plots with the gene sets significantly affected by the combination treatment compared to the anti-PD-1 treatment.
